# Orchestral Musicians' Community Engagement as a Means for Flourishing: A Phenomenological Study

**DOI:** 10.1002/jcop.70148

**Published:** 2026-09-21

**Authors:** Sara Ascenso

**Affiliations:** ^1^ Royal Northern College of Music Manchester UK; ^2^ University College London London UK

## Abstract

Recent decades have witnessed a rapid expansion of community music projects, and findings have highlighted their potential to build important psychological resources such as positive emotions, a sense of accomplishment, engagement, purpose and social skills. Despite the collaborative nature of this type of work, the experiences of the musicians who facilitate it remain largely undocumented. This study emerged from two innovative initiatives that brought together primary school children and musicians from two of London's major orchestras (London Philharmonic Orchestra and London Contemporary Orchestra) to work together to prepare and perform new music written by emerging composers. The aim was to investigate musicians' lived experiences of community engagement through these collaborations. Interpretative Phenomenological Analysis (IPA) was adopted as the research framework. Fifteen musicians (six females, nine males), ranging from 26 to 57 years of age, took part in one‐to‐one semi‐structured interviews after experiencing a month of community‐based work with the children. Data analyses revealed the experience of community engagement to be accounted for through three supraordinate themes, encapsulating 12 subordinate themes: (1) identity development (personal and collective), (2) well‐being (positive emotions, engagement, accomplishment, purpose and social contribution) and (3) skill development (personal, interpersonal, musical, teaching and creativity). The role of community engagement in fostering professional orchestral musicians' mental health is discussed.

## 1 Introduction

Music‐based community projects have been rapidly expanding in the classical music sector and now reach a wide set of contexts. A recent report by the Association of British Orchestras, with data from 53 UK orchestras, documented a yearly engagement of close to 700,000 people across community‐focused activities, including participatory sessions and performances (Association of British Orchestras 2025).

Community music refers to participatory musical practice that typically occurs outside of formal educational or professional performance contexts. It emphasises inclusivity, social engagement and life‐long learning through music, often valuing process over product (Higgins 2012; Veblen 2007). These local music‐making initiatives are varied in mediums, aesthetics and styles, and shaped by the cultural contexts they are embedded in (Bartleet 2023).

Alongside the proliferation of community‐based projects, there has been improvement in documenting the evidence of their impact. This includes research on their role in promoting psychological health at the individual level (Viola et al. 2023) in areas such as engagement, self‐esteem (Cohen et al. 2006; Davidson and Fedele 2011), a sense of accomplishment (Ascenso et al. 2018), agency (Ascenso 2021), purpose in life, control, autonomy, social well‐being (Cooper 2026; Creech et al. 2013) and social skills such as empathy and social awareness (Kirschner and Tomasello 2010). Active participation in the arts has also been highlighted as a means for developing a collective identity in communities (Smilde 2018). In a recent critical synthesis integrating 74 cross‐disciplinary papers, Bertleet and Heard (2024) go further in highlighting how community engagement with music can play an important role at a macro level in developing more equitable societies, both by addressing the causes of inequality and by mitigating its consequences. The authors call attention to equity outcomes at an individual level among disadvantaged communities, including well‐being, social connection, identity formation, self‐acceptance, reimagined futures, confidence and empowerment. Concurrently, community music engagement also supports individuals to enable equity‐related change through promoting critical reflection and consciousness, empathy, tolerance, pro‐sociality and the bringing together of diverse groups of people. Finally, these initiatives can encourage societal change more broadly, by promoting advocacy, positive social actions, interactions and visions of society, and by challenging social norms and attitudes (Bartleet and Heard 2024).

When assessing the impact of community‐based music projects, well‐being is frequently mentioned as an outcome. However, this often happens in a theoretical vacuum. Before describing the musical initiative under investigation, it is useful to situate this study theoretically and clarify its conceptual lens on mental health and flourishing.

Well‐being models have tended to emphasise either hedonic or eudaimonic dimensions (Keyes and Annas 2009). Within a hedonic perspective, wellbeing has been defined as the optimal balance between positive and negative affect along with perceived satisfaction with life (Diener 2000). Eudaimonic approaches, in turn, highlight virtuous action and self‐realisation, equating well‐being as the extent to which a person is fully functioning (Waterman 1993). This includes dimensions such as self‐acceptance, personal growth, autonomy, environmental mastery, positive relations with others, and purpose in life (Ryff 1989), as well as social well‐being (Keyes 1998). More recent proposals have included both hedonic and eudaimonic dimensions, equating well‐being as an integration of (1) emotional well‐being: positive affect and life satisfaction; (2) psychological well‐being, consisting of six dimensions: autonomy, environmental mastery, personal growth, positive relations with others, purpose in life, self‐acceptance; and (3) social well‐being, consisting of five dimensions: social integration, social contribution, social coherence, social actualisation, and social acceptance (Keyes 2002). Mental health is operationalized as a syndrome of the positive symptoms described above, and the term ‘flourishing’ is used to represent optimal levels across the three categories (Keyes 2002). In this study, we have adopted this framework when assessing the impact of community‐based music making. Besides being theoretically‐driven, this model has been empirically supported, both cross‐culturally and throughout the lifespan (Westerhof and Keyes 2010).

One of the components often highlighted in the assessment of community‐based music projects is the collaborative nature of music‐making and the mutuality underpinning it (Ascenso 2021; Higgins 2012). Echoing this, music institutions have turned away from traditionally calling this work ‘outreach’, denoting separateness from the community, opting instead for the terms ‘community engagement,’ or ‘partnerships’ to capture bidirectionality. Interestingly, however, research on the impact of community music initiatives remains focused on one side of this collaboration: the so‐called ‘participants’ of the projects. The experiences of the musicians who facilitate this type of work remain largely unaddressed.

Among professional orchestras, there has been some work seeking to understand how young musicians in the transition to the profession experience engaging with their communities. One example involved the Chicago Symphony Orchestra's Civic Fellowship programme, an early career scheme that includes performance, education and entrepreneurial musical initiatives, with a strong focus on developing leadership skills. It entails, for example, music creation among vulnerable groups. In the context of this scheme, a phenomenological study with eight young musicians revealed how community music‐making was experienced as a space for personal and professional growth (Ascenso et al. 2019). All participants reported this type of work as a catalyst for a new perspective on what it means to be a musician. Through experimenting different musical identities (performer, educator, community group leader, facilitator, entrepreneur), the musicians widened their view on the potential career paths music can allow and placed less centrality in performance in their concept of the profession. Several well‐being dimensions were also highlighted as a result of engaging with the local community. These included a sense of meaning, accomplishment, gratitude and hope. The study also underscored how community engagement constituted a fruitful space for skill development (e.g. entrepreneurship, versatility, creativity, teaching skills, musical skills, empathy, communication) and positive changes in self‐concept through greater self‐awareness and confidence.

If community music‐making is indeed a fertile ground for flourishing, and co‐creation is at the core of such initiatives, understanding musicians' experiences is key towards drawing implications about the ongoing proliferation of such initiatives in the music sector. Given the growing commitment to invest in community work, particularly by orchestras, a clarification on its potential for promoting flourishing among professional orchestral players is needed. Crucially, considering the opportunities community work opens to musicians for exploring new roles and different forms of engagement with music, it is especially appealing to understand the potential of this kind of work towards helping orchestral musicians navigate some of the psychological challenges their particular type of work brings. These include performance anxiety (Fernholz et al. 2019), a lack of engagement, loss of artistic input and boredom (Ascenso et al. 2017; Parasuraman and Purohit 2000). Concurrently, we also know musicians tend to report a high sense of meaning and purpose (Ascenso 2022; Ascenso et al. 2018), the musician identity as a giving identity and music as a relational space (Ascenso et al. 2017). The development of multiple identities of music‐making has also been highlighted as a route to greater well‐being among musicians (Ascenso et al. 2019; Ascenso et al. 2017). Engaging with communities in a highly relational initiative can, therefore, offer promise towards sustaining musicians' psychological health.

### 1.1 The Present Study

The starting point for this study was an initiative designed by the London‐based charity Music Masters, bringing together musicians from two of London's major orchestras, the London Philharmonic Orchestra and the London Contemporary Orchestra, alongside primary school children, to work together and perform new music commissioned to emerging composers. Music Masters is a London‐based charity founded in 2008, aiming to widen access to high‐quality music education throughout the lifespan, with a focus on disadvantaged communities, in order to contribute to building a truly diverse music industry. One of its key pillars of action involves working with state schools across the UK to model high‐quality and inclusive music education. Concurrently, Music Masters aims to champion, support and connect professional musicians, optimising their potential to bring positive change. The project was part of a series of initiatives with the goal of increasing orchestral musicians' contact with local communities with whom they would otherwise not typically engage with, while simultaneously supporting emerging composers in showcasing their work in respected concert venues.

Building upon these collaborations and on the musicians' previous experiences with community engagement, the aim of the present study was to understand how professional orchestral musicians make sense of their experiences of engaging with their community through collaborative music‐making, with a focus on flourishing, as defined above. In so doing and having in mind the recent expansion of these types of activities across the music sector, we anticipate to clarify if and how this work can bring opportunities for well‐being promotion among musicians as it does to the communities they are serving. This in turn can inform planning and ensure it is fully inclusive, optimising the benefits for all involved, while also changing the narrative from a one directional ‘giver‐recipient’ to one of co‐beneficiaries working with and for each other. To fulfil these aims, the research question guiding this project was ‘how do orchestral musicians experience a community engagement project in relation to psychological flourishing?’.

## 2 Methods

The project took place over 4 weeks and encompassed seven visits to primary schools, two rehearsals with the musicians and the children together, and a performance at the Royal Festival Hall in London's Southbank Centre. Musicians were involved in creating musical exercises with the children that were relevant to the preparation of the repertoire, led small group rehearsals in the schools, performed for the children and talked about their instruments and the music, and performed together at the final rehearsals and performance.

Purposive sampling was adopted. The criteria for inclusion were as follows: (1) to be a member of the project team as a musician; (2) to be part of all the project sessions and (3) to be fluent in English. All fifteen musicians engaged in the community project fulfilled the criteria, were invited and agreed to participate in the current study: eight members of the London Philharmonic Orchestra (LPO), three members of the London Contemporary Orchestra (LCO) and four musicians from the Music Masters team. The sample size is congruent with the upper range reported in IPA literature (Smith et al. 2009) and allows for case‐by‐case idiographic depth while enabling maximum representation and analytic coherence (Cena et al. 2024).

The group comprised of six women and nine men, aged from 26 to 57 years, from three specialisms: strings, woodwind and brass, holding between 4 and 35 years of professional experience as a musician. Demographic characteristics are presented in Table 1.

**Table 1:** Participant information.

Participant number	Age	Sex	Section
1	42	M	Strings
2	31	M	Strings
3	57	M	Woodwinds
4	29	F	Strings
5	32	M	Strings
6	48	M	Brass
7	35	M	Brass
8	28	F	Strings
9	32	F	Strings
10	41	F	Woodwinds
11	48	F	Woodwinds
12	31	M	Strings
13	28	M	Strings
14	27	M	Strings
15	26	M	Strings

In order to answer the research question encapsulating the richness and subjectivity of each participant's experiences (Cena et al. 2024), semi‐structured one‐to‐one interviews were conducted by the lead researcher with all musicians. They lasted, on average, 55 min and ran during the week that immediately followed the final performance. The interview questions were organised around three areas: (1) personal history of community engagement (e.g. ‘Before focusing on our project, let's talk about your journey as a musician so far. Have you had other community‐based experiences as a musician before? If so, please tell me about them.’); (2) individual experience of the primary school project (e.g. ‘thinking now about this project, how did you experience it overall?’) (3) the meaning of music‐based community work for musicians (e.g. ‘if you were to be invited to a panel discussion with other musician colleagues on the meaning of this kind of project, what thoughts would you like to share?’). Alongside the interviews, the researcher was part of the projects' rehearsal sessions and concerts, which allowed for reflective notes about the projects' characteristics, aiding in the contextualisation of the interview material. All interviews were recorded with permission and transcribed verbatim.

Interpretative Phenomenological Analysis (IPA) was adopted as the framework for the project, given its idiographic emphasis, prioritising the understanding of the participant's experience, in context (Eatough and Smith 2008; Smith et al. 2009). IPA places a focus on accessing each participant's emic perspective, with the researcher fulfilling an active, interpretative role in a double hermeneutic – making sense of how the participant makes sense of their experiences (Smith et al. 2009).

The analysis proceeded case‐by‐case, following five steps and making use of NVivo 11 software. After all interview material was fully transcribed, transcripts were closely read multiple times for familiarity and a holistic view of the data. Second, emergent meaning units were annotated on the left‐side margins for each transcript. The meaning units were then clustered together based on similarity to form emergent subordinate themes. Fourth, for each participant, the subordinate themes were integrated into tables. Descriptions for each of the themes and comments on their connections were noted. A table of themes was built for each participant. After close scrutiny of each transcript, individual tables of themes were integrated into one overarching table, representing the study's final supra‐ordinate and subordinate themes (see Table 1). Emergent themes were regularly revisited within and across transcripts to ensure a valid representation of the raw data. Codes and themes were cross‐checked by an external independent researcher to ensure accuracy and validity (Smith et al. 2009; Yardley 2008).

The guidelines for rigour in IPA research suggested by Nizza et al. (2021) were followed throughout every phase of the study. The focus was on building a robust account of the participants' experiences, supported by rich data extracts. A close analytic reading of the transcripts was ensured, and both convergence and divergence in the data were attended to. In keeping with the tenets of IPA, knowledge was understood to be co‐constructed through a double hermeneutic: my sense‐making of participants' sense‐making. I sought transparency, coherence, and context sensitivity, acknowledging that my perspective is integral to the interpretative process, rather than separate from it. Before the project, the interview schedule was carefully worded and tested in a pilot. During the interview, the key content and emotions were frequently reflected back to the participant to guarantee unbiased interpretations. To ensure rigour and that interpretation didn't happen in a theoretical vacuum, the construct of flourishing as is conceptualised by Keyes (2002) informed the analysis.

This project follows the standards for reporting qualitative research (SRQR) suggested by O'Brien et al. (2014) and was granted ethical approval by the Conservatoires UK Research Ethics Committee (CUK REC). All data were treated as strictly confidential. Identifiable information was removed during transcription and data were securely stored on password‐protected, encrypted devices accessible only to the researcher and in accordance with institutional data management protocols. All participants provided written informed consent. No compensation was given in exchange for participation.

### 2.1 Researcher Positionality

I carried out this study as both a psychologist, well‐being researcher and a trained musician, identities that shape how I understand the relationship between music‐making, community, and well‐being. My psychology training and clinical work have informed my interest in how individuals construct meaning from experience, while my work with music within diverse community settings has given me an insider appreciation of its emotional and social significance. Throughout the research process, I sought to remain reflexive about this positioning, acknowledging that my perspectives could influence the kinds of questions I ask, data interpretation and the meanings I construct. To address this, I maintained a reflexive journal during data collection and analysis, with notes about my interpretation which were thoroughly discussed with an experienced IPA researcher who acted as a consultant for this project and who also audited all its phases. My focus throughout was to ensure that participants' voices and meanings were represented authentically.

## 3 Results

Musicians' experience of community engagement was accounted for through three main themes: (1) identity development, (2) well‐being and (3) skill development. Each superordinate theme is built upon constituent subordinate themes representing different facets emerging from the data (Table 2). This section explores each superordinate and subordinate theme through example quotes that encapsulate their meaning.

**Table 2:** Superordinate and subordinate themes.

Superordinate theme	Subordinate theme	Descriptor
**1. Identity development**	1.1 Musical identity	The individual's comprehensive sense of musical self.
	1.2 Societal significance as a musician	Awareness of the societal significance of musicians’ professional class and of oneself in it.
**2. Well‐being**	2.1 Feeling Good	Hedonic well‐being
	2.2 Functioning Well	Eudaimonic well‐being
**3. Skill development**	3.1 Personal Skills	Skills related to individual regulation.
	3.2 Interpersonal Skills	Skills involved in interaction.
	3.3 Musical Skills	Skills involved in creating, performing, understanding, and/or appreciating music.
	3.4 Cognitive Skills	Skills related to information processing and problem‐solving.
	3.5 Teaching Skills	Skills that foster meaningful learning.

## 4 Identity Development

Community engagement was experienced as a contributor to musicians' awareness of who they are and the role they play in society, shaping their definition, beliefs, and expressions of what it means to be a musician, both individually and collectively. Two sub‐themes built the theme of identity: community engagement as a source for developing one's own sense of musical identity and community engagement as a source for enhanced connection with society leading to a greater awareness of the societal significance of being a musician.

### 4.1 Identity: Musical Identity

Participants highlighted community work as a building block for developing their musical identity through six pathways: (1) a clearer perception of the power of music; (2) a sense of being grounded, through providing a ‘reality‐check’; (3) new insight into musicians' contribution towards the benefit of the next generation, (4) a space for giving to others and an increased awareness of the sharing nature of music‐making; (5) the development of self‐expression through higher freedom in music‐making and (6) allowing to re‐visit personal narratives memories from their own past experiences with music.

First, community engagement facilitated a strong awareness of the power of music, a process often mediated by the enhanced personal interactions that a small setting allows, eliciting direct and constant feedback about music‐making.

It takes you back to what music does and it reminds you in a very real way of the power of music and the unique way it can stimulate people's lives and to be reminded of that as a musician when you're thinking about money, schedules, family life, work‐life balance, travelling… […] it is really enriching […] maybe if you've been doing too many concerts, this work reminds you of what it is you're actually doing… so when you play to all these people anonymously, what you're actually doing is speaking to them on an emotional level […] I think it is a very good thing for a professional orchestra musician to be reminded that you've taken someone on a really special journey!.

(Participant 6)

Closely linked with a clearer perception of the power of music, was the space for a ‘reality check’ on why musicians do what they do. Participants referred to this as feeling more ‘grounded’.

Quite a good reality check! […] As a high‐level professional musician, you can lose touch with the baseline and why you're there. […] It's a very useful exercise for people who maybe have been in the profession for a while because it keeps you grounded. It distils it to the very basic level… if you're playing and you see a child's face light up you can see that you're communicating with them on an emotional level… purely emotional and real, it's really very positive and uplifting to remind yourself… maybe if you've been doing too many concerts…

(Participant 6)

The musicians experienced community engagement as a space to combat the possible alienation that an intense career immersed in performance can imply, through greater awareness of the communicative power of music and a reminder of why they do what they do.

Thirdly, community engagement brought an opportunity to develop the sense of guiding the next generation. We borrow the term *generativity* from Erikson (1950) to encapsulate the meaning emerging from the data. This refers to the concern for establishing a legacy and nurturing the next generation, stemming from a sense of optimism about humanity. Participant 11 shared: ‘I just think it's important to educate the next generation of what you have learned. It's the most important thing and the most valuable thing you can pass on.’ Similarly, Participant 5 highlighted, ‘you're seeing the future of music in these projects…’. In this context, one participant shared a story of a student who, as a child, had been inspired by a community project he had facilitated and decided to become a professional musician. A decade later, this student unexpectedly met the musician again, this time sharing his stand at a major concert with a UK orchestra. Overall, musicians' experiences revealed community projects as a fertile ground for a stronger sense of purpose tied to the greater awareness of one's legacy, which seems to be harder to develop only through performances when a direct contact with the audience is not possible.

Community engagement was also experienced as a space for giving to others and developing awareness of the shared nature of music‐making. Giving emerged simultaneously as a definer of the profession – i.e. the musical identity equated as a giving identity – and as a much‐needed process to help deal with the industry's tendency towards self‐centredness. As Participant 9 expressed,

It's easy to lose sight of what benefits you are bringing to the world… For many musicians it's like ‘oh, I envy doctors’, that's a very tangible way of changing the world and for us… we're performing a concert. Ultimately a concert is that much of a selfish experience […] you are serving your ego ultimately. […] So this work is a reminder that you can go a step further and actually do something much greater for people. It feels like you're immediately changing the future for the better.

Musicians' experienced a sense of meaningful contribution through their work and reinforced that their profession can bring positive change, which seems to be especially important among classical musicians who can be seen as simply serving a self‐centred agenda of elite‐level performance.

A fifth aspect of experiencing community engagement highlighted in the context of a stronger sense of identity was self‐expression ‐ greater freedom and being allowed to express more of one's individual voice. Underlying this theme was a sense of ‘letting go’ of discipline, perfectionistic concerns and the worry of being under constant scrutiny, as well liberation from anonymity and the need to be led, embracing individuality and autonomy. Participant 14 developed on this theme as follows:

A lot of the time orchestral players hide behind their part. They hide behind their desk, they play the notes. […] you don't have to be an individual to be in an orchestra but to work with children, you have to be an individual… it's a challenge but it's freeing! […] Having to question how to be… because it does take some letting go… […] Prior to me getting involved with these projects, I was always too conscious. I'm a perfectionist therefore in my work and my performance, I need everything to be spot on […]. So when you understand that you just have to let go and stop being that… […] all of a sudden you do need to find some room for mistakes, and just be yourself. So that's very powerful!

Community music‐making appears as a much‐needed space for exploring being a musician without the extremely high pressure for perfection imposed by the classical music sector.

Finally, in the context of strengthening one's personal identity, musicians mentioned community work as a source of recalling their own individual journeys with music. The workshops evoked memories from early musical experiences, allowing them to re‐visit important personal narratives.

### 4.2 Identity: Societal Significance

Alongside developing one's musical identity, community engagement strengthened musicians' connection with society and was consistently mentioned as a pathway for increasing awareness of the profession's societal significance. In other words, this work was experienced as a source of several avenues for societal bonds which in turn grew awareness of the societal significance of the professional class and of oneself in it. In this context, community engagement was perceived by musicians as a source for 1) developing new audiences; 2) employability and 3) shaping music education.

The contribution towards developing new audiences was built upon the realisation that allowing community members a more direct experience with music increases their music appreciation and the value they attribute to live performance. This point emerged in relation to the growing accessibility of music online and the danger this represents to live audiences' sustainability. Another common thread was the opportunity for higher inclusion that community engagement work offers to the music sector:

What can we do to encourage people to listen more? I think it's a central thing for people to be engaged. Thinking of that engages me and to the same extent the workshops did as well, because it's like you're young musicians and you're loving it and potentially you could be the […] the audience of tomorrow, or the music lovers of tomorrow and that's really good and it's so different from everything I do, so it gave me a refresh about who our audiences actually are.

(Participant 12)

For the majority of participants, community engagement increased a sense of employability in a profession where many struggle to find work. The portfolio nature of music careers was also mentioned along with the need for broadening one's experiences across different facets of music‐making. As Participant 2 highlighted, ‘the reality is not everybody can play or perform as their job because there aren't any jobs. So it only seems sensible to me to be as rounded as you possibly can be, and if you can do some of this work that's another strength to you and a new possibility for work.' Doing this type of work was experienced as a way to broaden one's possibilities for being employable, in a sector where the diversity in social spaces to which musicians can contribute is increasing.

Finally, musicians highlighted how community engagement projects feed into the music education agenda, through allowing for role models who facilitate music learning:

we go on tour so much, and you just get the feeling that music education is so much more important, a journey, and just like that people are just much better educated with music […]. So to get any sort of hands on experience, or to be in an orchestra, or playing in an orchestra, or have orchestra musicians in the classrooms, I just think it's really, really important from an educational point of view. And obviously it makes you think about what you're doing yourself as well.

(Participant 2)

The experience of strengthening one's sense of contribution and societal significance through community engagement also involves a sense of helping the music education agenda and through it, musicians also think about and enhance their own approaches to music‐making.

## 5 Well‐being

Community music‐making was also experienced as a means for well‐being both through (1) feeling good, by increasing positive emotions and (2) functioning well, through engagement, a sense of accomplishment, purpose and social well‐being.

### 5.1 Well‐Being: Feeling Good

The facet of well‐being linked with pleasurable experiences and feeling good (hedonic well‐being) was consistently highlighted. Musicians found joy in doing these activities for their own sake but also through observing the joy they brought to the people they were working with. All participants referred to community work as uplifting: ‘You get a lot of satisfaction and enjoyment out of it’ (Participant 8). Gratitude was also highlighted, in two ways. First, musicians were impacted by the thankfulness coming from the people benefitting from projects: ‘you get a lot of gratitude back and of course that feels great’ (Participant 6). Secondly, an enhanced experience of personal gratitude for their own life circumstances emerged when they were introduced to the challenging life stories of the vulnerable populations they were working with: ‘It makes me feel lucky to be where I am. To be involved with what I'm involved in and I love, […] life in general can be tricky and you see ‐ you're faced with that. If you have grown up in… I would say… in a bubble, and you haven't experienced many bad things, you've been privileged to have a really good life. Then you're faced with problems that you never had to face as a child in these scenarios… actually it makes you appreciate what you've got which quite often we forget' (Participant 14). Gratitude was a central part of how every participant made sense of their experience of the project, with the richness of its relational encounters promoting appreciation in ways that the regular performance activity does not.

### 5.2 Well‐Being: Functioning Well

Musicians shared how community projects helped them not only feel good but also experience a sense of (1) engagement, (2) accomplishment, (3) purpose and (4) social well‐being.

Engagement refers to the sense of involvement in an activity. This was consistently highlighted in relation to the newness of the tasks musicians experience in community settings and also to how this work brought variety to their lives, fostering an increasing interest and helping with the challenge of routine often experienced in the orchestra.

If you don't set yourself these new challenges […] then you can't really complain that your life is a routine! […] So it's a matter of being open to doing something different in order to keep yourself interested in the normal part of the job […] I just think it helps me, for sure. I have to ask myself… I'm 42 now and I've got a great job and I like it, but it's likely that if I don't do anything different that I'm going to be sitting there in the next 20 years and doing similar things… how do I get through this or how do I keep interested in what I'm doing? […] the repetitiveness of the job is sometimes difficult to overcome!

(Participant 1)

Engagement was also mentioned in relation to greater variety in one's career more broadly. In the words of Participant 7, ‘It just widens your professional scope in a sense, and horizons, and you can just have a more varied career as a result really… because playing in the orchestra all the time I think would be quite difficult… Lots of people do other things, whether it's chamber music or teaching but I haven't done that so this is giving me another interest in terms of my career.’ There was a sense across all accounts of a desire to diversify the nature of one's professional music‐making activities to avoid stagnation, to enhance enthusiasm and novelty, and crucially, to experience a sense of progression and growth. Participant 5 also reinforced this theme:

I think a lot of people talk about retraining, and it is because it is quite stagnant. There's nowhere to go, it's just a question of getting enough work, and for that to be your career for the next 20 years when you've already done it, I think it's difficult to work with. Like in a normal career you know people are always thinking about where you can develop your skills, it's almost a requirement isn't it? […] I find [an orchestral career] very limited in terms of development for each individual. I feel like there's nothing more I can get. Of course I enjoy the concert, but in terms of what we do every day, we're not stretched. So just getting out, getting out of what you do on a regular basis and sort of seeing something else…

Reaching out to communities was also mentioned as a source of a sense of accomplishment and reward. This happened both through perceiving what one achieved via the workshops personally, but also by observing how other people benefitted from them. This well‐being component was often linked to the accounts of the greater awareness of the power of music, already explored under the section on identity (superordinate theme 1): ‘you do get a very heart‐warming experience from it when you do see these kids who've really developed during the project and that's very rewarding!’ (Participant 6). In the words of Participant 15: ‘it was one of those things… it was a lot of hard work but you come to the end of it and you think, ‘wow I was able to do that!’. I think if something like that was thrown at me again I'd know how to do it. […] I came home from certain sessions and it's got me: ‘yes’! You know? ‘That worked really well’.’

Participants also referred to community engagement as fostering a sense of purpose. This theme relates to the broader sense of individual meaning attributed to the profession already explored under the section on identity. However, purpose in this context appears mentioned with a very clear link to overall well‐being, beyond the scope of a musical identity. Musicians highlighted how this type of work can help develop a sense of belonging to something larger than oneself: ‘it's a sense of being more than just an average human being… yeah… And for me, it's quite important because I guess we're still young… we're radical, we're still thinking ‘what is the meaning of life… what's this all about…’ […] when you actually do something that…. you like, through giving to others, it feels there is a purpose, you know?’ (Participant 10).

All musicians seemed to be transformed by the realisation that through doing what they love and are good at, they were bringing value to their community in a very tangible way: ‘I think certainly it gives you a sort of sense of doing something valuable, when you go and you can see how it can change someone's life…[…] to come here and say ‘okay there is certain value in what I do’, you can sort of forget that…’ (Participant 5).

Crucially, musicians experienced community engagement both as a source of purpose, strengthening their reasons for engaging in their professional activity, but also as a source of significance, boosting their perception of the value of that activity.

Finally, community engagement was mentioned as a source of social well‐being.

I really think it is important, because [the orchestra] is a very insular world and we are very much confined. We often don't see past a second violin section or the first one and so I think in that sense it could certainly be beneficial to see or to take a project from the start to completion and be involved with other people.

(Participant 5)

Besides fostering more interaction, this project also brought a sense of engaging socially with a more diverse group of people and of enlarging one's social sphere in a meaningful way.

There's this whole other world of people that you could get to engage with on a different level. It's great, I think it's important to have both… for me personally, to have that balance.

(Participant 11)

Alongside a higher sense of connection with the external world outside one's professional music sphere, the musicians also experienced enhanced connection within their own orchestra: *‘Through these projects I've also got to meet and got to know some names and members of the orchestra that I wouldn't otherwise have known actually’* (Participant 4).

Finally, one of the musicians highlighted community engagement activities as a possible way of dealing with the social challenges that may arise within the orchestra's professional context:

We're not made to think about how to develop as a section, or how to work better, and there are a lot of personal problems. I think that in a modern business, it would be dealt with in a much better way, and that's a real problem. So it's quite refreshing to go into a new situation to a school and to do something that we almost take for granted and to sort of take it further.

(Participant 5)

Working in a community setting was seen as a fertile ground for developing transferable relational resources that can help deal with the orchestra's everyday interpersonal challenges.

## 6 Skills

The final group of emergent themes concerns skill‐development. Community engagement was experienced as a forum for acquiring and/or consolidating skills, as well as for a higher awareness of skills. This was experienced across five categories: 1) personal, 2) interpersonal, 3) musical, 4) cognitive and 5) teaching skills.

### 6.1 Skills: Personal Skills

The label ‘personal skills’ is used here to refer to competencies that are more related to individual traits than to specific job tasks. They feed into general functioning both within and outside of the work context. Three personal skills stood out across the data: 1) flexibility, 2) readiness and 3) understanding of child psychology.

Musicians highlighted the importance of flexibility in responding to the challenges brought by community engagement:

the ability to be doing music in a room where you've got very different types of learners […] and having a way of teaching the same material but tailoring it to those different needs in a group. You learn to be incredibly flexible […] you also develop an openness to learn other techniques from other colleagues […] and step back from your usual way of doing things sometimes.

(Participant 15)

This was often mentioned in relation to its subsequent generalisability to other contexts of life. The data suggests flexibility was developed as an emergent response to the structural demands of community teaching, including the need to hold multiple pedagogical approaches simultaneously, in real time, within a single group. It was also not experienced only as a teaching‐related skill, but as a broader disposition of adaptability.

The second personal skill mentioned was readiness, both general ‐ the ability to act quickly – described as being willing to step up in an activity or deal with unexpected behaviours, and also in relation to performance ‐ being able to play music on the spot, often with little preparation and without ideal conditions. Participant 9 noted: ‘you sort of break down that wall of, you know, you can react immediately – you're forcing yourself to immediately concentrate. […] It's perfect practice for when you actually have to walk on stage, but it's not about being just calm. I would say… […] what I developed was how to access a place of concentration despite distractions.’

The repeated need to respond immediately to unplanned situations functioned as a form of implicit training for readiness, a skill musicians also directly linked to their usual performance contexts. Importantly, participants distinguished readiness from mere calmness. Readiness was experienced as a skill honed through repeated exposure to unpredictability, rather than just an emotionally regulated state.

Finally, a third personal skill strongly highlighted was an understanding of child psychology, and a consequent easiness in relating to children. Community work brought an invaluable exposure to a rich set of behavioural patterns. Participant 13 pointed:

the number one thing for me is the child psychology thing […], that is something that you start to grasp. That makes it kind of a lasting thing, it isn't a musical thing, it's really just this kind of greater understanding of child psychology and just how to behave around children and how to treat them. To get the best out of them which just then leads you to just be better at acting with children around any situation.

Particularly strong within this theme, was the reinforcement of how this skill can be useful in other contexts. Some musicians highlighted the family setting, others the context of the orchestra in relation to effective concert programming. Participants explicitly framed this skill as a transferable form of relational competence. This was a strong instance of musicians making sense of their community engagement as a personally transformative experience, rather than only professionally relevant.

### 6.2 Skills: Interpersonal Skills

The development of skills linked with interaction was equally emergent from the data. These were as follows: (1) perspective, (2) empathy, (3) teamwork and (4) communication.

Perspective was mentioned with two meanings. First, in relation to understanding others: community engagement as a fruitful space for exercising one's understanding of different perspectives (applied to both children and colleagues). This is closely linked with the theme of flexibility mentioned earlier, however framed within the context of interaction. Perspective was also mentioned as the re‐definition of one's own problems and the re‐equation of their weight in the light of the broader picture that develops as a result of community work, linked with the gratitude sub‐theme described earlier. As Participant 14 expressed:

it puts things into perspective… […] actually it makes you appreciate what you've got which quite often we forget. And also… in a performance, because I get obsessed… So if I have a competition, I'll be practising 10, 12 hours a day, I mean, I'm just making myself ill to be perfectly honest with practice and that's not just me, musicians do tend to do that. Because we're workaholics but actually when you take a step back and think about the important things and actually focus, it makes you focus on the right things even within your performance. Makes me analyse things that I do and the way I perform and what I worry about and what I should worry about which are two different things most of the time.

The data suggests perspective arose from the contrast between the community context and the intensity of solo performance culture and values. Participants explicitly described a cognitive shift (‘when you take a step back’), evidencing how community work prompted an active re‐evaluation of what deserved attention or worry, which in turn also benefitted their performance routines.

Linked with perspective, some participants highlighted community work as a means to developing their empathy: ‘it certainly makes you more empathetic and so that may well feed into the way I perform. I found working with [name] was absolutely fascinating, that made a real impression on me because I realised ‘blimey! that could be me!’ and that was very thought‐provoking and made me much more benevolent in my attitude toward people who are down on their luck’ (Participant 6).

There was a sense of closeness and of a shared humanity established between the musicians and the participants that was often evident by musicians' comments on personal narratives about one's history or that of close relatives that the participants reminded them of, denoting empathy and compassion.

A third skill mentioned was teamwork, both in relation to fellow colleagues as well as children participants. The need for intentional training of teamwork in professional orchestras was highlighted: ‘we need to train teamwork. There are modern businesses that run workshops, courses, etc. A lot of my friends work in a wide variety of jobs, and they get on training courses once a month in these sort of skills. And they're valuable… they go away with their team of people and do something together. Why is it different with an orchestra?’ (Participant 5). Participants identified the structural absence of deliberate social skills training in orchestras, suggesting that community engagement can act as a compensatory, informal route towards the intentional training in this area seen in other professional sectors.

The project also enabled a forum for development of communication skills. In this context, musicians highlighted good strategies for a clear presentation of ideas: ‘it makes you develop a kind of clarity of thought that you can then turn into teaching, which is being able to present an idea clearly, however simple it is. Communication skills ‐ we have to talk a lot, because otherwise the children talk [laughs]. But within that, you still got to be able to teach something very clearly' (Participant 13).

Additionally, musicians stressed their community experiences as a prolific space for engagement with the orchestra's audience. The opportunity for developing public‐speaking and presentation skills was recurrently mentioned, as well as knowing how to communicate with all types of public: ‘it's very important to do these projects especially if you perform. I do a lot of solo repertoire and I find these community projects help me to understand how you engage with the audience. So when I perform, I like to talk to the audience, I like to tell them a little bit about the piece and how I got around to doing it and I just feel… in talking to people in the workshops, it opens up that possibility to make sure you're doing it in the right way […] and making sure there's a conversation with the audience’ (Participant 11).

The constraints of delivering a community workshop, with the pressure to hold children's attention, helped train clarity of thought and expression, which then generalised into musicians' broader teaching and communicative practice. At the same time, the community setting seems to have functioned as a rehearsal space in which conversational strategies for audience engagement could be tried out and refined before being carried into performance contexts.

Overall, engaging with the community was experienced as a space for relational dynamics seen as a much‐needed complement to the workplace experience as an orchestral performer.

### 6.3 Skills: Musical Skills

Participants also experienced working in the community as a means to develop musical skills. In this context, there were four areas of development: 1) going ‘back to musical basics’, 2)'dissecting' the musical pieces, 3) improvisation and 4) musical communication.

Working with the community brought musicians the opportunity of going ‘back to basics’‐ exploring the fundamental pillars of technique and expression: ‘you go back to thinking more about what you're doing […] which is something in the orchestra you may not do… you just go back to thinking about how to play things’ (Participant 7).

Regular orchestral practice can become proceduralised or habitual to the point that foundational elements of technique and expression are no longer consciously attended to. The demands of community work interrupt this automaticity, encouraging musicians to return to conscious, deliberate attention to technique and reactivate awareness of foundational skills.

This type of work also steered musicians towards a new understanding of musical material through the need to ‘dissect it’ in the process of making it more accessible to the communities they worked with. As Participant 11 highlighted:

it's very interesting to dissect the piece because we were performing it the next day and I just felt a lot more comfortable […] that's a very valuable thing… Because you're not just staring at the score, you are creating a different world based on the score, so you are getting ideas from the people that never heard it before. […] You dissect things in a different way, sort of in more detail.

The skill of ‘dissecting’ a piece developed through two combined pressures brought by the community context. First, the urgency of preparing for performance with very short rehearsal time, which demanded a close and detailed engagement with the score done quickly. Second, the work with a group encountering the material for the first time, whose reactions appear to have functioned as a novel source of interpretive feedback. Together, these shifted the musician's relationship to the score from straightforward reading toward a more generative way of engagement, ‘creating a different world based on the score’, rather than simply reproducing it.

The opportunity for developing improvisation was also thoroughly emphasised, linked with the theme of readiness mentioned before. The theme also emerged in relation to the idea of letting go of the need to ‘get it right’, moving out of one's comfort zone, as well as the importance of seeing music‐making in community as a process, in contrast with the focus on a perfect, finished, product. Participant 6 put it this way:

in terms of my own personal development there's one very significant thing and that's that […] you have to put the printed music to one side […] be prepared to improvise, and to react to what's being improvised around you, be it from another professional or somebody who maybe is bashing a drum for the first time. When you've been classically trained you are very, very hidebound by the process of reading music, following the composer's directions, giving the most perfect performance, it's all about perfection, idealism… It's tremendously useful to be pulled away from that discipline, to have to oh my god improvise, you know that's really scary, and the first time you do that, my goodness me are you out of your comfort zone! So it's almost the antithesis of what we do […] and it's all about the process rather than the product. Whereas when you're performing to a very high level it is very, very much about the product.

Improvisation is an activity that most orchestral musicians do not engage with in their professional context. The musicians in this project experienced a sense of apprehension at first for not feeling ready for the task. However, it soon became a valued and enjoyable experience. The focus on the process versus the finished product was especially meaningful as it represented a shift from their usual *modus operandi*.

Finally, the theme of communication re‐appeared but framed as a musical skill. Working with the community stimulated musicians to think about how to communicate musically with different groups and enhance their translation of musical ideas:

I find that performing in those situations is actually a really beneficial thing for me because you can communicate in a different way and that's actually made me realise that that kind of playing is the kind of playing I really enjoy a huge amount… playing with a community audience. It kind of makes you play in a different way. […] If you're doing a workshop, not only do you learn specifically how to talk about music, but also perform music to children… The way you perform […] might be different to a group of children to how you would play in the [concert venue in London], for example. That might also set thoughts rolling for ‘ok how could I play here… oh maybe I might be interested in doing this in a care home or a homeless shelter’ or something like that… just kind of acknowledging the way we present music can be different for different groups so… through doing that kind of work it would help to develop those types of musical skills.

(Participant 15)

It seems that musical communication was developed through the richness of encounters that community engagement allows, exposing musicians to audiences with different needs, modes of engagement and responses. This required musicians to diversify not just what they played but how they played. As participants described new potential contexts for this type of engagement, they seem to have moved beyond just in‐the‐moment adaptation towards a more generative skill, suggesting the development of a transferable schema for tailoring musical delivery to diverse audiences. Of note is also the mention to enjoyment in this context, indicating that communication wasn't simply improved through necessity but reinforced through positive affective feedback. This created a self‐sustaining loop where more communicative playing felt rewarding, encouraging further development of the skill rather than being experienced as a compromise on the musicians' usual performance norms.

### 6.4 Skills: Cognitive Skills

Working in new environments and with new musical tasks was highlighted in relation to cognitive skills. Linked with community music as a space for enhanced self‐expression and freedom, and also in relation to the opportunities for improvisation, musicians stressed the potential for developing 1) creativity and 2) focus.

The opportunity to perceive in new ways, to build novel approaches, to make new connections and generate new solutions, were part of the experience of community engagement. This emerged in contrast with the repetitiveness that can arise after several years in an orchestra:

for an average orchestra musician who plays the same orchestral works year in and year out… the creativity's gone a long time ago. Sometimes I wanted to just throw a grenade in the centre of [location] because after you've been playing the same Tchaikovsky ballet for the last 15 years, there's nothing creative about it. […] So the benefit of this work is obvious!

(Participant 10)

It stood out that there seems to be a paradox around classical music performance: it is part of a so‐called creative industry; however, it remains a business where there is little room for experimenting with novel ideas and conformity to tradition is reinforced.

To a smaller extent, community work was also mentioned as potentially helping enhanced concentration. Participants highlighted how workshop settings enable focusing on the most important elements during a task and the development of strategies to avoid distractions.

### 6.5 Skills: Teaching Skills

The final area of musicians' skill‐development enabled by community work relates to teaching. Participants highlighted the development of their ability to manage a group and gain new pedagogical resources, alongside being inspired by colleagues and learning through observation.

You sort of learned how to deal with a class and sort of make it fun. Trying to get them to come up with their own ideas. It's very easy to say ‘ok you'll do this’ and obviously you know what you're doing whereas if you were actually… if you try and draw it out of them, or if you can see there isn't much going on just say ‘well have a go at it’ or ‘what kind of stuff do you like…’ and trying to sort of control it and trying to get them not to play their instruments as well is also very tricky… so all this management of the group.

(Participant 8)

Despite some musicians having some teaching experience, their context of teaching is typically on a one‐to‐one basis and with elite young musicians. This project brought an opportunity to develop group teaching skills with beginners, enabling not only effective group management strategies but also engagement with entry level music pedagogy processes. When mentioning the ability to deal with a class, leadership as a core skill was also highlighted: ‘*you definitely develop leadership basically in terms of being a lead tutor and kind of managing a group of children for example’* (Participant 15).

Musicians also experienced community work as a source for gaining new, useful, resources for teaching. While some mentioned new ways of addressing specific music‐related content, others highlighted general strategies and a better understanding of the teaching process more globally:

there are several things I picked up along the way like certain little things you can do to make the child quiet instantly, little tricks… the bow on the head, getting them to hold their instrument in a certain way, getting them to line up in a certain way, just things like that to go through the basics… how to teach the rest position… I think it's very helpful because it instils positivity and just to think of it in a different way. I learned a lot of these things.

(Participant 12)

Musicians experienced the development of teaching skills less as a formal instruction and more as informal, incidental accumulation, with strategies ‘picked up along the way’.

In summary, experiencing community projects has the potential to strengthen orchestral musicians' sense of identity, both through a greater awareness of oneself and of the profession's societal significance. These projects also represent a prolific ground for increasing well‐being, through enabling positive emotions and promoting engagement, a sense of accomplishment, purpose and social well‐being. Finally, community projects serve as a forum for developing new skills, or consolidating existing ones, including personal, interpersonal, musical, cognitive and related to teaching.

## 7 Discussion

This study sought to understand the experience of community engagement of fifteen orchestral musicians in relation to psychological flourishing. The findings highlight the positive role this type of activity can play in strengthening musicians' musical identity, a sense of societal significance, key elements of well‐being and skill development.

The results from the first overarching theme of identity invite us to consider the complexity of the professional pursuit of music‐making. Musicians often negotiate complex professional roles and experience tensions between artistic integrity, authenticity and institutional expectations (Ascenso et al. 2017; Burland and Davidson 2004). It is striking that a group of musicians employed in one of the best European orchestras reports reconnecting with their musical identity and the societal value of music‐making *outside* of their day‐to‐day professional musical performance routine. This strengthens the case for orchestras to invest in broadening the contexts and human encounters for collaborative music‐making they provide to their musicians, enabling to bridge the gap audience‐musician often felt in typical performance situations, as well as fostering musicians' opportunities for creative leadership and artistic decision‐making (Ascenso et al. 2017; Ascenso et al. 2022). A crucial thread emerging from the data in this context is how community engagement promoted self‐expression through higher freedom in music‐making and brought an enhanced sense of purpose and significance. Previous research has pointed to meaning as a particularly strong pillar to a sense of well‐being among musicians (Ascenso 2022; Ascenso et al. 2018). Musicians often enter the profession with high sense of meaning, often describing music‐making as central to their identity (Dobrow 2013). However, institutional orchestral cultures may arguably weaken meaning over time, through a lack of space for musicians to have an individual voice and contribute artistically (Ascenso 2022; Parasuraman and Purohit 2000). Community engagement may therefore help musicians restore coherence and regain alignment with their original motivations to pursue musicianship, through promoting creative experimentation, idiosyncratic expression, freedom and a direct experience of music's impact.

Musicians also voiced how community engagement strengthened their awareness of the profession's societal significance. The project provided a sense that what they do matters, both on an individual level and as a professional class, and made them feel valued and appreciated for their contribution. Research has highlighted a high sense of social contribution among musicians (Ascenso 2022). Yet, at the same time, there can be a perception among artists of societal devaluation in relation to one's art (Barker et al. 2009), suggesting that while musicians may feel they have an important contribution to make to their communities, they can also be faced with a perception of limited appreciation of that contribution from society. The experiences of this study's participants suggest that community engagement work offers a space that strengthens *both* a sense of contribution and a sense of societal appreciation.

Secondly, community work contributed to musicians' well‐being, not only through providing pleasurable moments, but also by fostering key eudaimonic components such as engagement, a sense of accomplishment and social connection. This is particularly encouraging when considering the challenges associated with orchestral musicians in previous research, such as routine and boredom (Ascenso et al. 2017; Parasuraman and Purohit 2000), competition, debilitating perfectionism and performance anxiety (Fernholz et al. 2019). Community projects may therefore function as a buffer to some of the challenges brought by orchestral professional music‐making. This kind of work provides a space where musicians are given new musical challenges and are encouraged to lead in artistic decision‐making. This can reignite engagement amid a typical orchestral season, where often repertoire is familiar and repetitive. Working with the community, musicians also find a space where there is no competitive pressure to perform at the highest level, and where experimentation is encouraged and the process is largely more important than the product.

Finally, engaging with community projects brought these musicians a very structural contribution towards their growth, through serving as a laboratory for experimenting and consolidating skills. This was transversal to very diverse areas of functioning and included personal, interpersonal, musical, cognitive and teaching skills. Embracing community work as intentional professional development training in the context of orchestras gains here strong support. Of particular interest is the theme of creativity. In a sector self‐endorsed as a ‘creative industry’, there have been questions about how much space is given to classical musicians, especially performers, for developing creativity in their job. Leech‐Wilkinson (2020, 2022) has brought to light how music‐making is often haunted by strict performance norms and interpretative constraints and how this can impact musicians, especially through thwarting their creative input. Community engagement projects offer a platform for agency, freedom and self‐expression, empowering musicians to develop their individual voice and appear therefore as an important pathway to counterbalance the potential rigidity of a performance job. Parasuraman and Purohit (2000) found that a lack of artistic integrity was among the most potent stressors for orchestral musicians. The authors went further in noting that authoritarian leadership styles in orchestral settings, paired with a lack of participation in decision‐making, result in many musicians feeling unseen and undervalued and with an underutilized skillset. The results of the current study bring to light how community initiatives can serve orchestral musicians also in these areas by encouraging the use of a wide set of skills and welcoming artistic leadership and agency. Once again, this helps frame community engagement as a process that may counterbalance structural constraints often present in orchestral settings.

For music institutions, the experiences shared by the musicians in this study open up the discussion on how this type of work may move from peripheral outreach activities to becoming firmly considered as an ongoing part of the professional musician's portfolio. Especially taken into account its impact on well‐being and its potential as a laboratory for building transferrable skills, community engagement could be formally recognised as part of musicians' professional development initiatives.

This is a time when arts funding is under scrutiny and orchestras are investing in demonstrating music's societal contribution to strengthen their public relevance (Association of British Orchestras 2025). Oftentimes this is when community work is highlighted. However, besides reinforcing the strong case around reaching wider audiences, this immersion in communities may also start to be highlighted by institutions as a way to support their musicians' growth, creativity, risk‐taking and well‐being and in so doing, contribute to their career longevity. Engaging together with the local community can also build a better relational climate among orchestra members themselves, fostering cohesion, which in turn has the potential to impact orchestras' internal cultures. Future research will allow to continue to clarify not only how to best plan community initiatives from both the participants' and the musicians' point of view, ensuring that the design of a project optimises flourishing for all involved, but also how this type of work impacts individual performance and organisational‐level outcomes for orchestras.

The results of this study also inform conservatoires on the value of including community engagement into their curricula, not just for community‐based pathways of training, but for all musicians‐in‐training. Not only can this work help prepare musicians for portfolio careers beyond performance, it may also foster skill development and a widening of professional identities and well‐being.

### 7.1 Limitations and Further Research

The current study remained focused on one community and on a small sample of musicians. This scope was needed for an in‐depth, idiographic investigation of musicians' experience, in context. Our findings invite now further research that expands to different orchestral cultures and to community projects among other populations. The use of multi‐site designs, enabling comparison analyses, will allow to assess applicability to other settings, verify if the main areas of impact are replicated, as well as identify potential transferable mechanisms.

In addition, large‐scale surveys across orchestras engaging in community work across time, would help assess change sustainability. A longitudinal design may also clarify potential moderators and mediators in the relationship between community engagement and well‐being outcomes. From the results of the current study, one variable of interest is the level of autonomy each project allows the musicians to have in their work. Performing artists are mostly absent from organisational psychology research (Delle Fave and Kocjan 2016). The results of this study support the need to further investigate the dynamics of workplace well‐being in artistic professions, especially in what concerns the role of creative autonomy as a potential mediator between work structure and musicians' well‐being.

Despite participants being assigned to the project by the orchestras, there is still a possible self‐selection bias permeating this study. The pool of musicians within the orchestras who showed availability may already consist of those who are more motivated, socially oriented, or open to these experiences. Further research would benefit from comparison groups with an investigation among musicians not involved in community engagement. Where feasible, a random assignment would be valuable.

Despite our assurance of anonymity, there is still the possibility of a positive reporting bias. The musicians may have emphasised community engagement benefits due to social desirability or the orchestra's expectations surrounding the project. Community work may involve emotional labour, time pressures and other challenges that were not fully explored. Enlarging our methodological tools for triangulation, using observation and diaries to capture real‐time fluctuations in well‐being, will be of value to mitigate this.

In summary, the communities musicians work in play a vital role in their flourishing. In a time where orchestras invest more in so‐called ‘outreach’ initiatives, this process needs to be better understood in order to do justice to the mutuality it implies, seeing musicians and community groups as co‐beneficiaries, working with and for each other. The present study provides evidence that community engagement activities for orchestral musicians are not peripheral to their lives and growth but rather stand as psychologically and professionally transformative, with the potential to reshape their experience of identity negotiation and connection within their careers, well‐being and skill development. The engagement, purpose, creativity and connection contrast with, and may be compensating for, the routinised, competitive and constrained nature of orchestral performance routines. These findings suggest engagement with communities may be crucial for musicians' psychological flourishing.

## Ethics Statment

This project was granted ethical approval by the Conservatoires UK (CUK) Research Ethics Committee.

## Conflicts of Interest

The author declares no conflicts of interest.

## Data Availability

The data that support the findings of this study are available on request from the corresponding author. The data are not publicly available due to privacy or ethical restrictions. The ethics protocol required to ensure participants that only the final findings and anonymised quotes would be publicly shared and that permission would be sought should the data be used for other purposes.
